# From Carrier Effect to Collagen Remodeling: Toward Endpoint‐Specific Longevity Assessment in Polycaprolactone‐Based Collagen‐Stimulating Fillers

**DOI:** 10.1111/jocd.71204

**Published:** 2026-09-28

**Authors:** Chuan‐Yuan Lin, Jui‐Yu Lin

**Affiliations:** ^1^ Nanzi Annyeong Clinic Kaohsiung City Taiwan; ^2^ Taipei Royalty Aesthetic Clinic Taipei City Taiwan; ^3^ Chengdu Royalty Aesthetic Clinic Chengdu City Sichuan Province China; ^4^ Hangzhou Royalty Aesthetic Clinic Hangzhou City Zhejiang Province China

**Keywords:** biostimulatory filler, collagen‐stimulating filler, longevity assessment, neocollagenesis, polycaprolactone, three‐dimensional imaging

Dear Editor,

The growing use of polycaprolactone (PCL)‐based collagen‐stimulating fillers has intensified interest in treatment longevity [1, 2]. Yet, “longevity” is often used as if it represents a single measurable outcome. In PCL‐based fillers, early visible correction, maintained clinical improvement, objective volumetric change, and tissue‐level remodeling are related but not interchangeable [1, 2, 3]. Although the immediate carrier effect and later collagen‐mediated remodeling of PCL‐based fillers have been described [1, 2, 3], the implications of this sequence for endpoint selection and time‐window‐specific assessment remain insufficiently separated. To address this gap, we propose endpoint‐specific longevity assessment as a practical interpretive framework, not a validated outcome instrument.

PCL‐based fillers provide a useful example because available evidence spans clinical, imaging, and tissue domains. Controlled clinical studies have demonstrated sustained nasolabial fold improvement using validated scales such as the Wrinkle Severity Rating Scale and Global Aesthetic Improvement Scale [1, 4, 5]. These outcomes are clinically meaningful; however, they primarily describe patient‐ or investigator‐rated correction rather than the amount of tissue volume created or maintained. In contrast, three‐dimensional (3D) imaging can provide objective volumetric information. In a Vectra 3D case series, measured midface volume was maintained at 24 months and, in some patients, exceeded the initially injected volume [2]. However, persistence of surface‐derived volumetric change does not establish the underlying biological mechanism. Complementary data further support this biologic pathway, with ultrasound demonstrating dermal thickening and histology showing neocollagenesis after PCL injection [3].

On this basis, Table 1 presents a proposed tri‐phasic framework for time‐specific endpoint interpretation. In the early carrier‐dominant phase, apparent volume is influenced by the carboxymethylcellulose carrier, immediate contour change, and procedure‐related edema [1, 2, 6]. Accordingly, safety, tolerability, and early swelling are more relevant than durability. During the transitional phase, carrier dissipation and edema resolution may make reassessment more reliable, and staged dosing may be considered when clinically appropriate. Finally, in the late remodeling phase, durability claims should be anchored to the endpoint being measured: clinical scales for fold correction, 3D imaging for volumization, and ultrasound or histology for tissue remodeling [1, 2, 3, 4, 5].

**TABLE 1:** Tri‐phasic endpoint framework for longevity assessment in PCL‐based collagen‐stimulating fillers.

Phase	Approximate window	Dominant contributors	More appropriate endpoints	Main interpretive caution
Early carrier‐dominant phase	Days to weeks	CMC carrier, immediate contour change, procedure‐related edema	Safety, tolerability, early swelling, contour stability	Early volume should not be equated with durable correction
Transitional reassessment phase	Weeks to months	Carrier dissipation, edema resolution, early host response	Clinical reassessment, optional consideration of staged dosing, and adverse‐event monitoring	Premature retreatment may overcorrect or confound outcome evaluation
Late remodeling phase	Months to years	Collagen‐mediated tissue remodeling around PCL microspheres	WSRS/GAIS, 3D volumetry, ultrasound, biopsy/histology when ethically and clinically feasible	Clinical improvement, objective volume, and tissue remodeling should not be treated as interchangeable endpoints

*Note:* This proposed framework was derived from the reported timing of carrier resorption, resolution of early postinjection effects, and subsequent clinical, imaging, and tissue‐level assessments. It has not been prospectively validated. The phases are approximate, may overlap, and should not be interpreted as discrete biological boundaries. Their timing may vary according to formulation, dose, injection plane or depth, and host response.

Abbreviations: CMC, carboxymethylcellulose; GAIS, Global Aesthetic Improvement Scale; PCL, polycaprolactone; WSRS, Wrinkle Severity Rating Scale.

This temporal separation also has safety implications. In a large retrospective series of PCL‐based filler treatments, prolonged edema was associated with higher injection volume [6]. Delayed nodules and inflammatory reactions remain recognized potential complications, and management may be more difficult because PCL‐based fillers cannot be immediately removed by enzymatic injection [6]. These considerations should remain part of patient counseling and adverse‐event reporting. Therefore, early fullness should not be equated with durable correction, and persistent late clinical improvement should not automatically be assumed to prove objective volumetric replacement. For practitioners, the framework may aid patient counseling and follow‐up planning by distinguishing early fullness from durable correction, delaying reassessment until carrier and edema effects stabilize, and matching late longevity claims to the endpoint being measured.

Importantly, available evidence remains heterogeneous, and imaging and histologic data are still limited. The proposed framework therefore requires prospective validation using standardized clinical scales, serial 3D imaging, ultrasound, histology when ethically and clinically feasible, and standardized adverse‐event reporting. Future studies of PCL‐based collagen‐stimulating fillers should specify whether longevity refers to clinical correction, objective volumization, tissue remodeling, or long‐term safety. The question is not simply how long a PCL‐based filler lasts, but which endpoint is being used to define persistence.

## Funding

The authors have nothing to report.

## Ethics Statement

The authors have nothing to report.

## Conflicts of Interest

The authors declare no conflicts of interest.

## Data Availability

Data sharing not applicable to this article as no datasets were generated or analyzed during the current study.
